# The Reliability of the Divergence Angle for Evaluating Rotational Implant Positioning in Medial Unicompartmental Knee Replacement

**DOI:** 10.7759/cureus.42956

**Published:** 2023-08-04

**Authors:** Vincent Hardy, Marwan Garaud, Bandar M Hetaimish, Ramy Samargandi

**Affiliations:** 1 Orthopedic Surgery Department, Centre Hospitalier Régional Universitaire (CHRU) d'Orléans, Orléans, FRA; 2 Orthopedic Surgery Department, Faculty of Medicine, University of Jeddah, Jeddah, SAU; 3 Orthopedic Surgery Department, Centre Hospitalier Régional Universitaire (CHRU) de Tours, Tours, FRA

**Keywords:** knee surgery, divergence angle, rotational positioning, knee osteoarthritis, unicompramental knee arthroplasty

## Abstract

Background

Unicompartmental knee arthroplasty (UKA) is a highly effective surgical procedure used to treat patients with osteoarthritis affecting a single knee compartment. UKA has gained significant popularity, accompanied by an expansion of its surgical indications. This increasing trend can be attributed to the consistently excellent clinical outcomes associated with UKA, which rival those achieved with total knee arthroplasty (TKA). However, despite these advancements, implant rotation malposition remains a prevalent factor contributing to early failure in UKA cases. The aim of this study is to analyze the rotational positioning of femorotibial implants in UKA and to identify an appropriate angle formed by the femoral component and the tibial component using a newly described angle.

Methods

This was a retrospective study of patients’ data of 40 medial UKA cases of 33 patients who were operated on in our hospital between October 1998 and March 2019. The study introduces a new angle called the "divergence angle." This angle is formed between the lateral portion of the femoral component and the lateral part of the tibial component, as measured on a patellofemoral Merchant view at 30 degrees of knee flexion. The divergence angle was evaluated through radiographic assessment by two independent reviewers.

Results

According to statistical analysis, the divergence angle was highly reliable with both intra- and inter-observer reproducibility. Intra-observer reproducibility was excellent with an intra-class correlation coefficient (ICC) between 0.901 and 0.933 (p < 0.001). The inter-observer reproducibility was excellent with an ICC of 0.92 (p < 0.001). The Gaussian curve confirmed the normal distribution of the divergence angle values with moderate dispersion of values. The majority of the angles of divergence (85%) measured between the femoral and tibial components were less than 10 degrees (n = 34), with a mean angle of 6.3 ± 4.5°.

Conclusion

The divergence angle between the femoral and tibial components, measured at 30 degrees of knee flexion using the Merchant view, is an easily accessible, reliable, and reproducible method. This technique enables the assessment of the optimal rotational positioning of implants in medial UKA.

## Introduction

Unicompartmental knee arthroplasty (UKA) is a less invasive surgical alternative to total knee arthroplasty (TKA) that is used to treat patients with symptomatic knee osteoarthritis, where only one compartment of the knee is involved. It was introduced by McKeever in 1952 and its utilization has increased widely during the last three decades [1].

The knee after UKA is closely related to normal knee kinematics and is associated with low perioperative morbidity and intraoperative blood loss. Moreover, it allowed earlier mobilization and rehabilitation when compared with conventional knee arthroplasty [2]. 

Presently, approximately 8% of knee arthroplasty is performed using UKA in the United States [3]. UKA is associated with better clinical and functional results than TKA. Moreover, it has faster recovery rates, fewer complications, and reduced hospital stay [4-6]. Although UKA has a shorter lifespan than TKA, it is associated with excellent long-term and patient's satisfactory results [7,8]. A limitation of UKA is that a small percentage (1-3%) of patients may require future revision surgery, either due to the progression of osteoarthritis in the other knee compartment or implant loosening [5,8,9]. Improper positioning of implants can lead to premature wear and loosening of prosthetic components, necessitating early revision surgery [9-12]. Rotational malposition of implants, especially of the femoral component, is considered a factor contributing to early wear and consequently failure of the UKA. For instance, Assor et al. reported that the main reason for UKA failure is the rotational malposition of the femoral implant [13]. This could be overcome by improving instrumentations and using techniques to re-establish the three-dimensional alignment of the femoral component.

The success rate of UKA depends on several factors including patient selection criteria, age, sex, and level of activity [14]. Proper surgical technique and optimal implant positioning are paramount for obtaining satisfactory and long-term outcomes [15]. Therefore, major factors for achieving long-term and excellent outcomes involve accurate implant positioning and reconstruction of the lower limb mechanical axis [16]. Several previous studies have examined the rotational alignment after UKA using CT analysis [17-20]. However, relying solely on CT analysis for rotational alignment assessment can be limiting due to the associated cost, radiation exposure, and the requirement for specialized equipment. Interestingly, there is a noticeable scarcity of literature that evaluates rotation alignment using X-ray imaging.

This study assesses the rotational positioning of femoral and tibial implants in UKA by analyzing the divergence angle. The divergence angle refers to the angle formed between the lateral portion of the femoral metal component and the lateral portion of the tibial component, as measured on a patellofemoral Merchant view at 30° of knee flexion. It is worth noting that this angle has not been previously described or investigated. The study hypothesizes that measuring this angle could offer an easily accessible, reliable, and valid technique for performing UKA. The primary objective of this study was to establish the reliability and reproducibility of measuring the divergence angle.

## Materials and methods

Population

The study included patients who were operated on for a primary medial UKA between October 1998 and March 2019. The surgery was performed by several senior surgeons. The implants used in the surgery were HLS unicompartmental prostheses procured from the Tornier Laboratory or implants manufactured by them (HLS Uni, HLS Uni Evolution, HLS 2). Systematic radiographic follow-up by Merchant view was performed. We excluded all patients with a history of lower limb surgery, including fractures that may affect initial anatomical alignment, as well as patients who underwent high tibial osteotomy or had missing data, including the absence of a postoperative Merchant view.

Evaluation of the divergence angle

**Figure 1:**
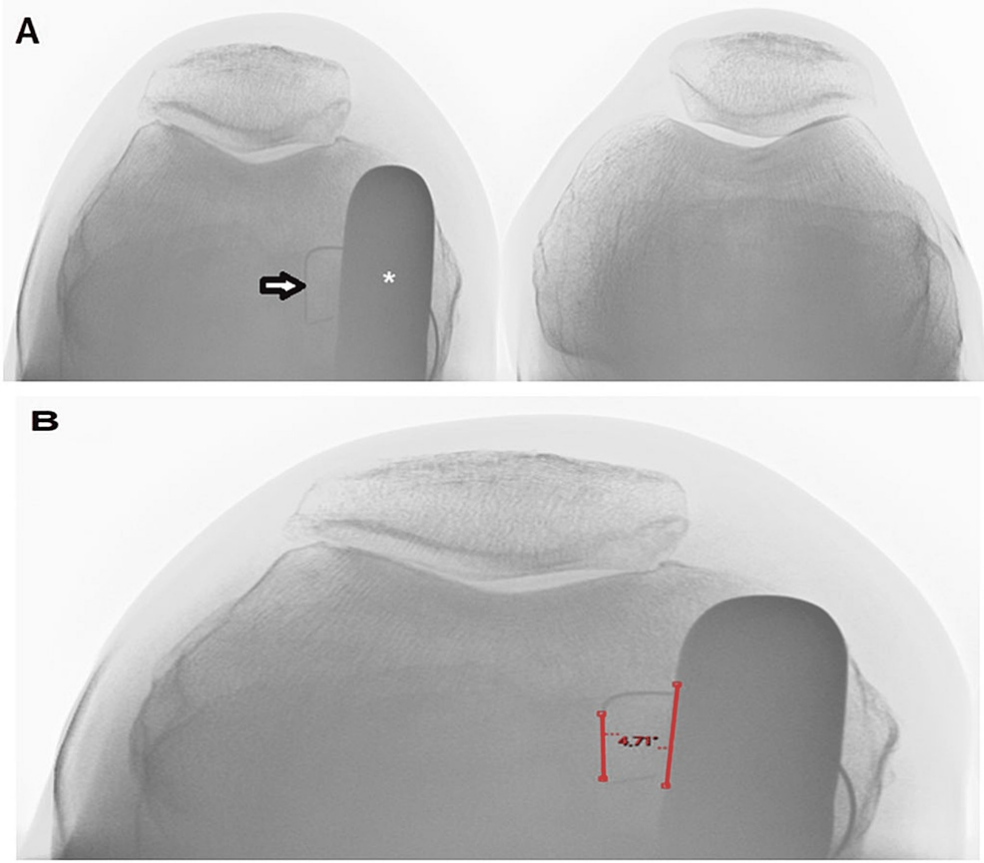
(A) Postoperative patellofemoral Merchant view at 30° of knee flexion, displaying the tibial component (arrow) and femoral component (*). (B) Measurement of the divergence angle between the lateral portion of the femoral component and the lateral part of the tibial component.

The divergence angle was evaluated through radiographic assessment by two independent reviewers (two fellows from our orthopedic surgery department). The radiographic data were collected using the PACS® software (Carestream Health; Canada, version 11.4.1.0324). The divergence angle between the femoral component and the tibial component was measured on a patellofemoral Merchant view at 30° of knee flexion, as part of the routine patient follow-up (Figure 1). A divergence angle objective of less than 10° was arbitrarily defined, allowing reproducibility of the surgical technique.

Each observer measured this angle on the Merchant view radiograph for each patient. It was ensured to analyze the radiograph of the best quality that provided the lowest angle of divergence. The first observer repeated the measurements five times, one week apart to assess intra-observer reproducibility. For the inter-observer reproducibility study, we used the first series of measurements from the first observer and the series from the second observer.

For the descriptive radiographic study of the divergence angle, we calculated the mean of the first observer’s five measurements for each patient. The dispersion of the values was shown by the construction of a Gaussian curve.

Statistical analysis

A normal probability plot on the EXCEL® software (Microsoft, United States, version 16.21) was created that enabled to analyze and confirm the normality and the Gaussian distribution of the values ​​for each of the series of measurements. Statistical analyses were performed by SPSS® (version 23; IBM Corp, Armonk, NY), XLSTAT® (Addinsoft, France, version 2019.4.2), and R® (The R Foundation for Statistical Computing, Vienna, Austria, version 3.5.2). Intra- and inter-observer reproducibility was assessed using Pearson’s correlation coefficient (r), the Bland and Altman diagram [21], and the intra-class correlation coefficient (ICC, ρ). The number of subjects to be included in the study was calculated according to the normal distribution method [22], which demonstrated the need to include 36 patients to have an ICC of 0.75, guaranteeing a lower limit of 0.5, an alpha risk of 0.05, and a power of 80%. The strength of the correlation was classified as strong (r > 0.5), medium (0.3 < r <0.5), weak (0.1 < r <0.3), or zero (r < 0.1) [23]. The strength of reproducibility was classified as excellent (ρ > 0.90), good (0.75 < ρ <0.90), moderate (0.50 < ρ <0.75), or poor (ρ < 0.50) [24]. We used a Wilcoxon signed rank test for paired data to compare the angle of divergence between the Merchant radiographic views selected for the study and other Merchant views in patient follow-up.

## Results

Population

The study included 40 medial UKA cases in 33 patients (22 females and 18 males), including 17 right knees (42.5%) and 23 left knees (57.5%). The mean age at the time of the surgery was 70.5 years ± 8.2 years (45-84 years). The mean follow-up was 85.2 months ± 63 months (8-238 months).

The intra- and inter-observer reproducibility of the measurement

For intra-observer evaluation of the measure of the divergence angle, the correlations between successive measures were strong and significant with Pearson’s coefficients r equal to 0.91 (measure 1 - measure 2), 0.90 (measure 1 - measure 3), 0.94 (measure 1 - measure 4), and 0.93 (measure 1 - measure 5) with p <10^−5^. Intra-observer reproducibility was excellent with ICC between 0.901 and 0.933 (p < 0.001) (Table 1).

**Table 1:** Means and standard deviations of the divergence angle measured five times by the first observer and intra-observer reproducibility. DA, divergence angle; ICC, intra-class correlation coefficient.

Measures n = 40	DA in ° (mean ± standard deviation)					Intra-observer reproducibility			
	Measure 1	Measure 2	Measure 3	Measure 4	Measure 5	Pearson	p-Value	ICC	p-Value
	6 ± 4.4	6.4 ± 4.4	6.1 ± 4.8	6.2 ± 4.8	5.9 ± 4.5	>0.9 (strong)	<0.001	>0.9 (excellent)	<0.001

For the inter-observer assessment of the divergence angle measure, the correlation was strong and significant with Pearson’s coefficient r equal to 0.92 with p <10^−5^. The inter-observer reproducibility was excellent with an ICC of 0.92 (p < 10^−3^) (Table 2).

**Table 2:** Means and standard deviations of the divergence angle measured by the two independent observers, and the inter-observer reproducibility. DA, divergence angle; ICC, intra-class correlation coefficient.

Measures n = 40	DA in ° (mean ± standard deviation)		Intra-observer reproducibility			
	Observer 1	Observer 2	Pearson	p-Value	ICC	p-Value
	6 ± 4.4	6,3 ± 4.8	0.92 (strong)	<0.001	>0.92 (excellent)	<0.001

**Figure 2:**
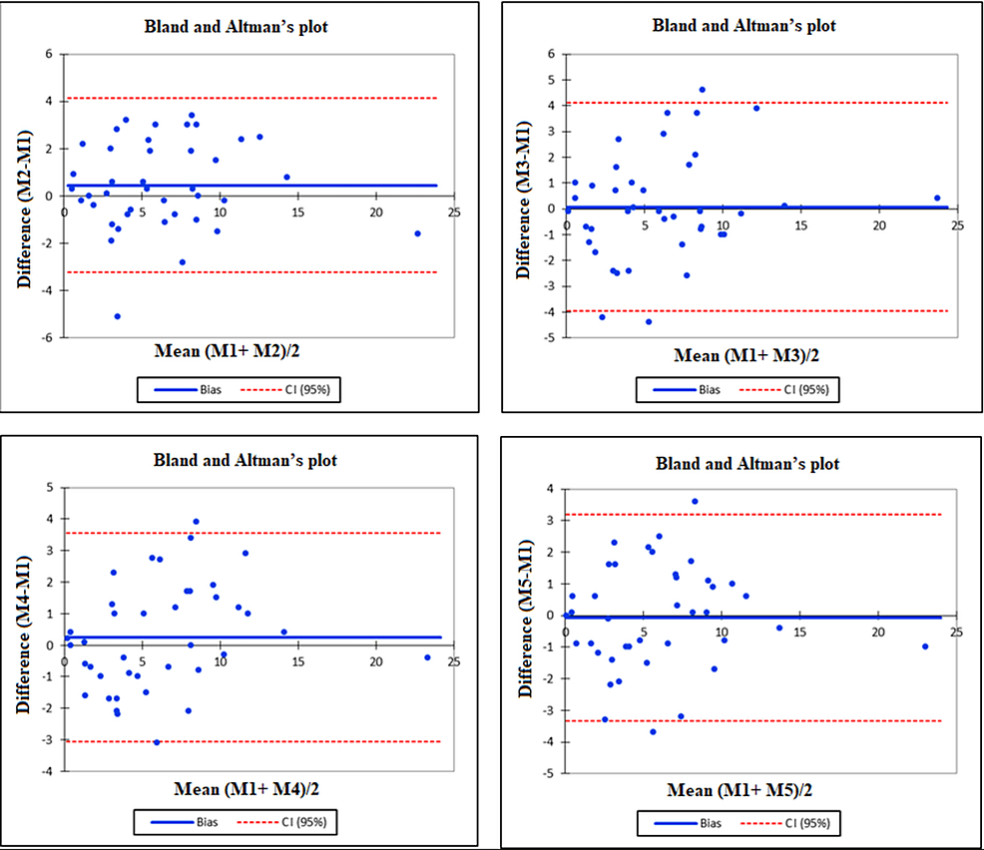
Bland and Altman's plots illustrating the concordance between the intra-observer measurements: (a) Measure 1 vs. Measure 2, (b) Measure 1 vs. Measure 3, (c) Measure 1 vs. Measure 4, and (d) Measure 1 vs. Measure 5. CI, confidence interval.

**Figure 3:**
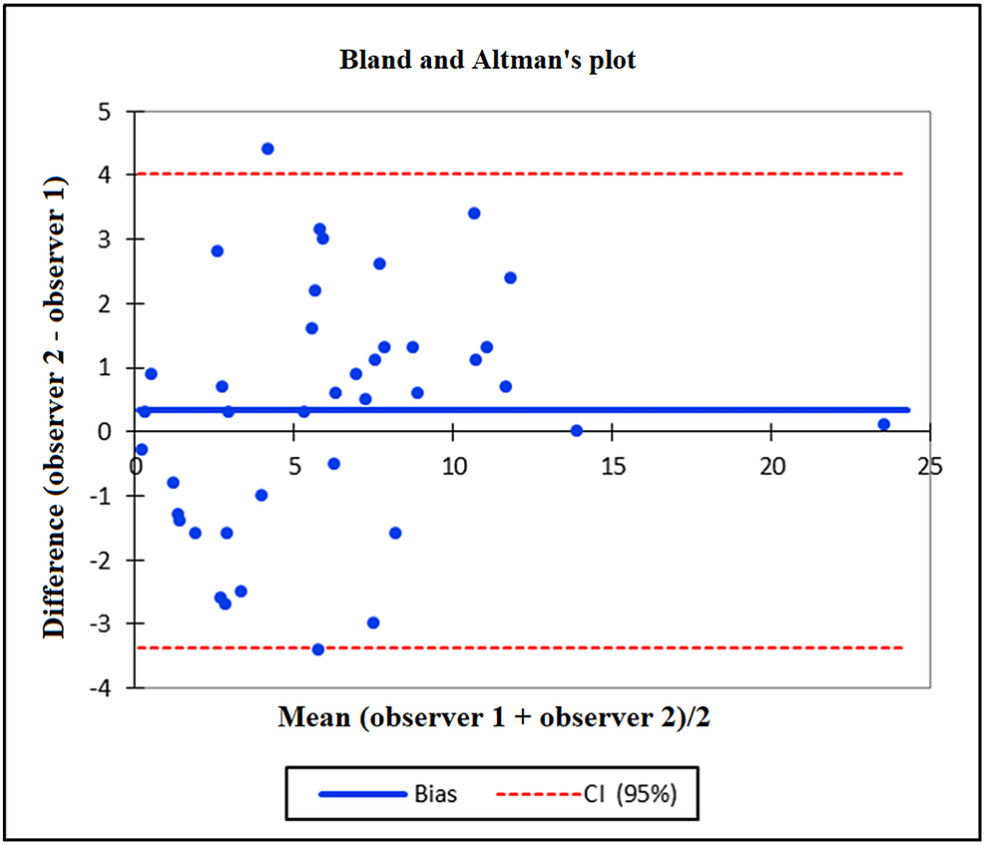
Bland and Altman's plot demonstrating the concordance between the inter-observer measurements. CI, confidence interval.

Further, Bland and Altman’s plot showed the concordance between intra- and inter-observer measurements of the divergence angle between the femoral component and the tibial component. The values were distributed equally above and below the zero line, within the range given by the upper and lower bounds of the agreement, with a few exceptions (Figures 2, 3).

**Figure 4:**
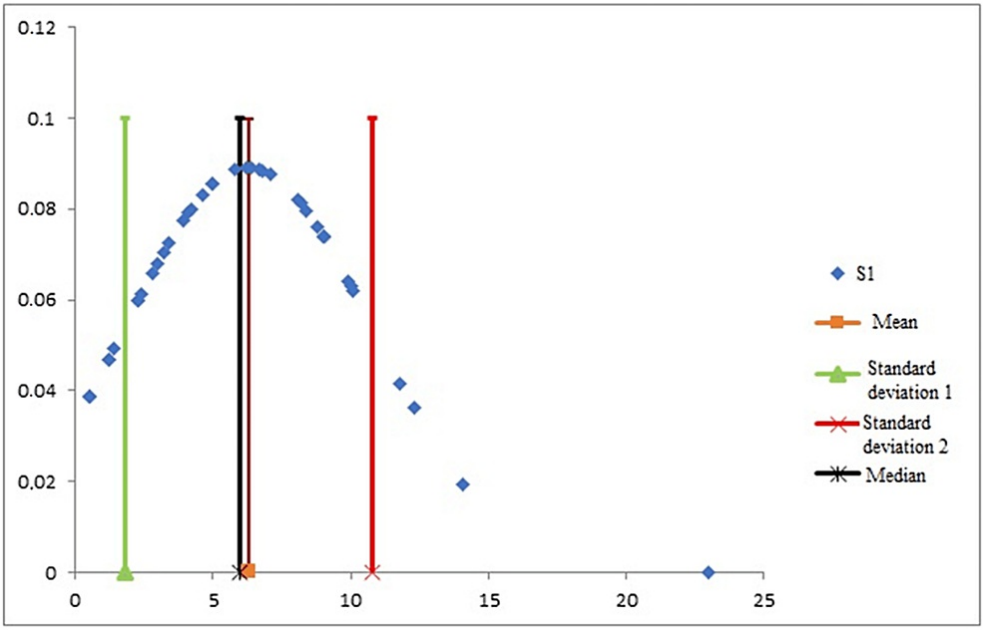
Gaussian curve confirming the normal distribution of the divergence angle values.

For the first observer, the mean divergence angle between the femoral component and the tibial component was 6.3° ± 4.5° (0.5-23), with an angle of less than 10° in 34 cases (85%). This result is illustrated by a Gaussian curve that confirmed the normal distribution of the divergence angle values and illustrated the moderate dispersion of the values. Most of them were within 1 SD of the mean and the median (Figure 4). The second observer recorded a mean divergence angle of 6.3° ± 4.8° (10° to 23.6°), with the angle being less than 10° in 33 cases (82.5%).

## Discussion

Results of the statistical analysis revealed important aspects of reliability with both intra- and inter-observer reproducibility of the divergence angle measured on the Merchant view. The divergence angle limit was arbitrarily set at 10° to study the rotational positioning of medial UKA to ease the radiographic inter-observer and intra-observer analysis. 

Most of the previous studies have used CT to assess the component alignment. For example, Servien et al. [18] studied the tibial component's rotational positioning in medial and lateral UKA with an analysis of two-dimensional CT reconstructions. The tibial plateau rotated laterally in the medial and lateral UKA, with an average rotation of 6.5 ± 5.1° in the medial UKA.

Lee et al. proposed other techniques to avoid the rotational malpositioning of medial UKA. They conducted a CT study that suggested the anterosuperior iliac spine (ASIS) as a landmark for the sagittal tibial cut in mobile-bearing UKA. Because of the rotational variations and the intraoperative identification difficulties of ASIS associated with the risk of iatrogenic injury to the posterior cruciate ligament in case of rotational malposition of the tibial component, the authors' recommendation was to avoid using this landmark technique [17].

Zhou et al. conducted a recent study in which they assessed the rotational alignment of the tibial component following UKA in 269 patients. The measurement of Akagi's line on CT scans was utilized for this evaluation, which is a line drawn from the axial image from the medial border of tibial tuberosity anteriorly to posterior cruciate ligament posteriorly [19,25].

Hence, there exists a requirement for an easily accessible, reliable, and reproducible low-irradiation measurement technique.

It is important to note that while most studies focus on assessing the position of each component in relation to its specific segment [17-19], our study describes an angle that considers the relationship between both components. It is essential to acknowledge that if both components are equally malrotated, the measurement will fall within the defined "10° limit." Although individual component evaluations provide valuable insights, our angle measurement offers a comprehensive assessment of the overall alignment in relation to the entire joint. By considering the interaction between the femoral and tibial components, we aim to provide a more holistic and quick view of the joint's rotational alignment.

Furthermore, the potential implications of this combined angle measurement on clinical outcomes are crucial. For example, if both components tend to be malrotated in a similar manner, it might affect the overall stability and functionality of the joint, even if the divergence angle appears satisfactory (<10°). However, we believe that this scenario is extremely rare since most component rotation in UKA is primarily attributed to the femoral component only [13].

This study had certain limitations like the retrospective design and having a small number of patients. Furthermore, the validation of this angle on clinical outcomes had not been examined. Another limitation is that the method adopted for taking Merchant view X-rays could be varying for each center and each radiological technician, which might introduce errors in the measurement. It is, therefore, essential to establish a standard protocol that allows capturing Merchant view X-rays in the same position and according to the same methods, thus introducing reliability in everyday practice.

Despite these shortcomings, the strength of this study lies in the evaluation of the measurement of this divergence angle on the patellofemoral view (Merchant). Interestingly, the measurement of this angle has not been described before; we proposed it in the study because it has several positive aspects. First, it is an easily accessible tool. The patellofemoral view (Merchant) is a part of the routine follow-up of patients who have undergone UKA. Second, it is a cost-effective technique. Third, it has a minimal radiation exposure since the X-ray radiations are considerably lower than that of CT scans.

The measurement of the divergence angle on the patellofemoral view shows promise as a practical method for assessing rotational alignment in UKA. Further research with larger patient cohorts is needed to compare different patellofemoral views and validate the advantages and limitations of this technique in clinical practice. Additionally, future studies should investigate the correlation between the measured divergence angle and the postoperative functional outcomes to enhance the understanding of its clinical significance.

## Conclusions

The newly described measurement method of the divergence angle between the femoral and tibial components in UKA demonstrates strong intra- and inter-observer agreement, highlighting the reliability of this technique. Our findings indicate that the divergence angle is a reliable and easily accessible approach for evaluating rotational alignment in medial UKA through radiographic analysis. However, further research involving larger cohorts is warranted to validate and explore the clinical significance of this technique or to compare its accuracy with CT scans.
